# Impact of Childhood Trauma on Stress Levels in Chronic Fatigue Syndrome Patients

**DOI:** 10.1192/j.eurpsy.2025.408

**Published:** 2025-08-26

**Authors:** Y. Dooms, M. Van Den Houte, I. Coppieters, E. Vergaelen, S. Claes, K. Bogaerts, L. Van Oudenhove

**Affiliations:** 1 KU Leuven, Leuven; 2 UHasselt, Hasselt; 3 VUB, Brussels; 4 Maastricht University, Maastricht, Belgium

## Abstract

**Introduction:**

Chronic Fatigue Syndrome (CFS) is marked by physical and cognitive fatigue, as well as increased susceptibility to fatigue. While the precise causes of CFS remain unclear, there is growing interest in the role of the stress response system in its development.

**Objectives:**

Given that early adverse experiences might affect one’s ability to handle stress effectively, the aim of this study was to examine whether such early life events could predispose patients with CFS to higher self-reported stress levels when confronted with psychosocial stressor.

**Methods:**

76 patients with CFS and 45 healthy controls (HC) underwent the Maastricht Acute Stress Test (MAST) to induce stress. Subjective stress levels were assessed before, during and after the task. The Childhood Trauma Questionnaire (CTQ) was used to retrospectively evaluate abusive and neglectful experiences in childhood

**Results:**

Patients with CFS reported significantly higher levels of subjective stress at all stages of the MAST compared to HC (main effect of group; p<0.0001). Additionally, CFS patients had higher CTQ scores than HC (p=0.04). Within patients, higher levels of childhood trauma was associated with higher levels of self-reported stress (p = 0.0047) throughout the MAST. The results of the link are visualized in the attached figure.

**Image 1:**

**Figure:**
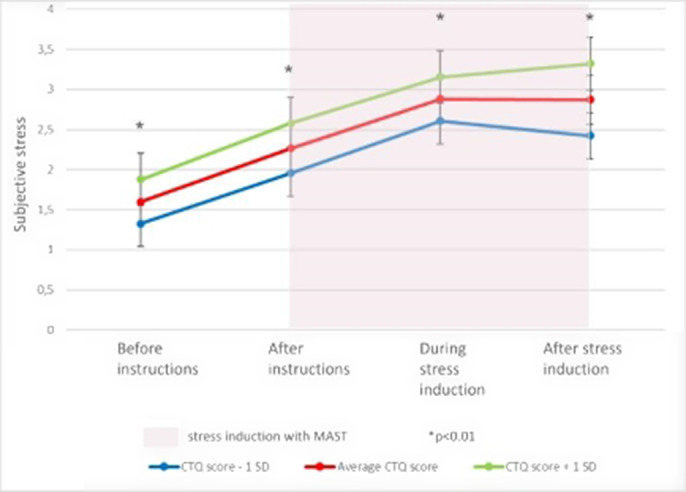

**Conclusions:**

Patients with CFS experience heightened stress levels during a validated stress-inducing task compared to HC. Furthermore, a history of greater childhood abuse and neglect is associated with increased stress levels later in life, potentially contributing to the development of CFS.

**Disclosure of Interest:**

None Declared

